# Angiographically borderline left main coronary artery lesions: correlation of transthoracic doppler echocardiography and intravascular ultrasound: a pilot study

**DOI:** 10.1186/1476-7120-9-19

**Published:** 2011-06-14

**Authors:** Zoltán Ruzsa, Attila Pálinkás, Tamás Forster, Imre Ungi, Albert Varga

**Affiliations:** 1 Department of Medicine and Cardiology Center, Faculty of Medicine, Albert Szent-Györgyi Clinical Center, University of Szeged, Szeged, H-6724, Szeged, Pécsi str. 4, Hungary; 2 Department of Internal Medicine, Erzsébet Hospital, Hódmezővásárhely, Hungary

## Abstract

**Background:**

the clinical decision making could be difficult in patients with borderline lesions (visually assessed stenosis severity of 30 to 50%) of the left main coronary artery (LM). The aim of the study was to evaluate the relationship between transthoracic Doppler (TTDE) peak diastolic flow velocity (PDV) and intravascular ultrasound (IVUS) measurements in the assessment of angiographically borderline LM lesions.

**Methods:**

27 patients (mean age 64 ± 8 years, 21 males) with borderline LM stenosis referred for IVUS examination were included in the study. We performed standard IVUS with minimal lumen area (MLA) and plaque burden (PB) measurement and routine quantitative coronary angiography (QCA) with diameter stenosis (%DS) and area stenosis (%AS) assessment in all. During TTDE, resting PDV was measured in the LM.

**Results:**

interpretable Doppler signal could be obtained in 24 patients (88% feasibility); therefore these patients entered the final analysis. MLA was 7.1 ± 2.7 mm^2^. TTDE measured PDV correlated significantly with IVUS-derived MLA (r = -0.46, p < 0.05) and plaque burden (r = 0.51, p < 0.05). Using a velocity cut-off of 112 cm/sec TTDE showed a 92% sensitivity and 62% specificity to identify IVUS-significant (MLA < 6 mm^2^) LM stenosis.

**Conclusion:**

In angiographically borderline LM disease, resting PDV from transthoracic echocardiography is increased in presence of increased plaque burden by IVUS. TTDE evaluation might be a useful adjunct to other invasive and non-invasive methods in the assessment of borderline LM lesions. Further, large scale studies are needed to establish the exact cut-off value of PDV for routine clinical application.

## Introduction

Coronary angiography is the gold standard procedure to assess the severity of coronary artery disease, despite of its well-known limitations [1]. Significant left main coronary artery (LM) disease, (defined angiographically as stenosis severity > 50%) of luminal diameter, is associated with poor prognosis when medically treated and usually requires coronary bypass surgery [2,3]. However, the clinical decision making could be difficult in patients with borderline lesions (visually assessed stenosis severity of 30 to 50%) of the LM. Intravascular ultrasound (IVUS) confers the ability to examine accurately the coronary artery architecture, atherosclerotic plaque composition and changes in vessel dimensions as a result of the atherosclerotic process [4-8]. Hence, IVUS is the preferred method to assess the severity of angiographically borderline LM lesions [9-13]. Although it provides detailed anatomical information on the vessel lumen, it is an expensive and invasive procedure. Several other diagnostic tools, such as myocardial perfusion scintigraphy [14,15], stress echocardiography [16], multidetector computed tomography [17] and coronary flow reserve measurement [18,19] have been proposed as valuable adjunct to coronary angiography in the difficult clinical decision making process of patients with borderline LM lesions. It has also been demonstrated, that simple resting Doppler transthoracic echocardiography (TTDE) is an effective method in assessing hemodynamically significant LM lesions [20-22]. Its diagnostic and prognostic effectiveness, however, is unknown in case of borderline LM stenosis. The present study was designed to evaluate the potential correlations between IVUS, quantitative coronary angiography (QCA) and TTDE in angiographically borderline LM lesions, and to determine, whether TTDE could have play a role in the decision making process.

## Patients and Methods

### Patient population

Consecutive patients with angiographically documented borderline LM stenosis (30 to 50%) (n = 27, mean age: 64 ± 8 years, 19 males) were enrolled in the present study. Following coronary angiography, LM has been evaluated by IVUS and TTDE in all patients. All patients with positive treadmill stress test and angiographically documented borderline LM stenosis entered this pilot study. Indications and contraindications of the angiography followed the coronary revascularization guideline of the European Society of cardiology [23]. Exclusion criteria were: hemodynamic instability, acute myocardial infarction, hypertrophic cardiomyopathy, severe obesity (BMI > 35 kg/m^2^), or known congenital heart disease. Patients were informed about the study itself, its proceedings, and possible adverse events. The study satisfied with Declaration of Helsinki and was approved by ethical committee of the University of Szeged.

### Transthoracic Doppler echocardiography

TTDE studies were performed with a Vivid 8 ultrasound equipment (General Electric, New York, USA) using a 3.5 MHz transducer with harmonic imaging. All TTDE studies were carried out by a single investigator experienced in LM TTDE assessment, blinded to the angiographic and IVUS results. B-mode image was used to identify the LM and pulsatile Doppler to measure the flow velocity in diastole. Imaging plane was oriented in parallel with short-axis view of the aortic root slightly above the aortic valve. Sample images were stored digitally for subsequent analysis.

### Quantitative coronary angiography

Hemodynamic procedures have been performed with an angiographic **s**ystem equipped with a digital flat panel imaging detector (Innova 2000; General Electric). All patients underwent standard coronary angiography, at the beginning of which intracoronary nitroglycerine (0.2 mg) was administered to achieve maximum vasodilatation. Measurements were then taken from two orthogonal views. Data were recorded on CD-ROM to allow off-line assessment. An experienced invasive cardiologist blinded to IVUS and TTDE findings performed off-line the QCA analysis. The edge-detection technique developed by Reiber (CMS-GFT, Medis (Leiden, The Netherlands)) was used for QCA assessment [24-26]. An empty guiding catheter was used for calibration, and diameter stenosis was determined using a quantitative analysis program (Centricity Cardiology, CA1000, General Electrics, USA). Stenosis was considered significant if QCA showed diameter stenosis greater than 50%.

### Intravascular ultrasound

IVUS was performed together with coronary angiography at one sitting using an Atlantis Plus 40 MHz catheter **(**Boston Scientific Inc., Natick-MA, USA). After cannulation of LM, a 0.014" guide wire was introduced into the distal LAD and an IVUS catheter was placed into the distal position. The IVUS catheter was withdrawn at 0.25 mm/s by automated pullback, while IVUS measurements were recorded on super VHS. Quantitative measurements were taken off-line according to the IVUS standards of the American Society of Cardiology [27]. In each case minimum lumen diameter (MLD in mm), minimum lumen area (MLA in mm^2^), external elastic membrane cross-sectional area (EEM-CSA in mm^2^), plaque burden (%) and area stenosis (%) were measured. A physician, who was experienced in IVUS, but independent of the study, measured vessel diameter at its narrowest region. LAD stenosis was considered significant if LCSA was smaller than 4 mm^2 ^[28], while stenosis less than 6 mm^2 ^indicated significant LM stenosis [11].

### Statistical analysis

Categorical data are presented with absolute frequencies and percentages, continuous variables as medians with interquartile ranges or as means ± standard deviation. Differences between continuous variables were analyzed by Student's t test. The relationship between the results obtained by QCA, TTDE and IVUS and was evaluated using Pearson's correlation rank test. Bland-Altman analysis was performed to test agreement between modalities. P < 0.05 was considered to reveal significant statistical difference. Statistical analyses were performed using the Sigma-Stat software package (San Jose, California, USA, Systat Software In.).

## Results

Clinical and demographic patient data are demonstrated in Table 1. All study patients had a positive treadmill test or had ST segment depression under chest pain.

**Table 1:** Clinical characteristics of study population

Parameters	n (%)
Age (year)	64 ± 8

Male gender (%)	18 (75)

Systemic hypertension (%)	16 (67)

Hypercholesterolemia (%)	14 (58)

Current smoker (%)	8 (33)

Diabetes mellitus (%)	7 (29)

Canadian angina classification:	
- I.	0 (0)
- II.	5 (21)
- III.	11 (46)
- IV.	8 (33)

Previous myocardial infarction (%)	9 (37.5)

Previous coronary bypass surgery (%)	0 (0)

### Transthoracic echocardiography

Interpretable Doppler signal could be obtained in 24 patients (88% feasibility).

### Coronary angiography

All patients had visually assessed borderline LM stenosis. Isolated left main disease was present in 14 patients, but coronary angiography revealed additional atherosclerotic lesions in the remaining 13 patients: 3 vessel disease in 2 patients, 2 vessel disease in 3 patients and one vessel disease in 5 patients. The summary of LM TTDE, QCA and IVUS measurements are demonstrated in Table 2.

**Table 2:** Quantitative coronary angiography, IVUS and TTDE findings

QCA	
- MLD (mm)	2.6 ± 0.7
- Diameter stenosis (%)	36.5 ± 9.6
- Area stenosis (%)	58.15 ± 12.25
- Reference diameter (mm)	4.10 ± 1.10
- Lesion length (mm)	7.2 ± 3.2

**IVUS (quantitative analysis)**	

LM MLA site	
- MLA (mm^2^)	7.1 ± 2.7
MLD (mm)	2.75 ± 0.7

### Intravascular ultrasound

Clear IVUS images were obtained in all cases and no complications occurred during the recordings. IVUS revealed significant LM stenosis in 17 patients (71%) Fourteen patients with significant LM stenosis underwent percutaneous coronary intervention (PCI), 3 patients were referred for bypass surgery, while 7 patients were considered having hemodynamically insignificant lesions, and therefore were recommended for further medical treatment and follow up (29%).

### Correlations between angiographic, IVUS and echocardiographic parameters

**Figure 1:**
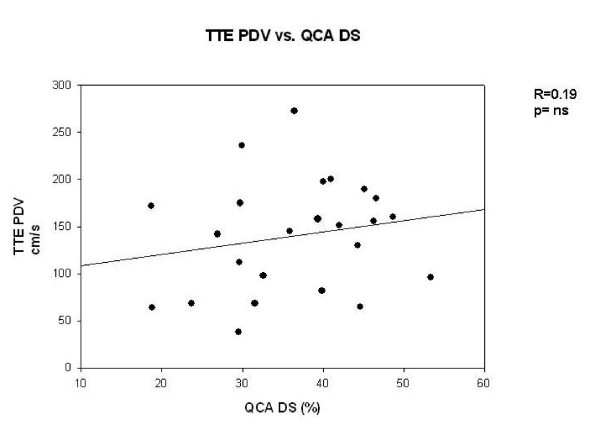
**Comparison of peak diastolic velocity (TTDE) and diameter stenosis (QCA) show no significant correlation**.

**Figure 2:**
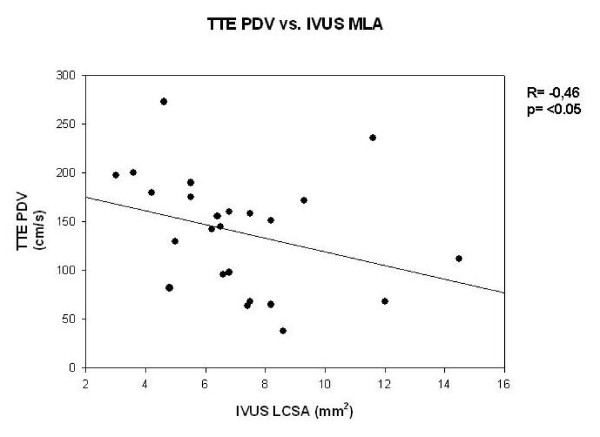
**Comparison of peak diastolic velocity (TTDE) and minimum lumen area (IVUS) show significant correlation**.

**Figure 3:**
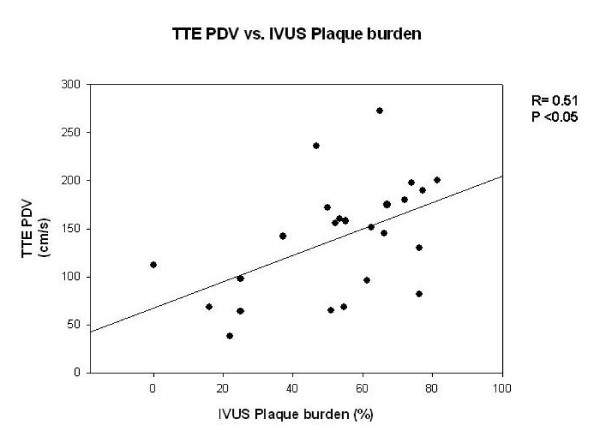
**Comparison of peak diastolic velocity (TTDE) and plaque burden (IVUS) show significant correlation**.

**Figure 4:**
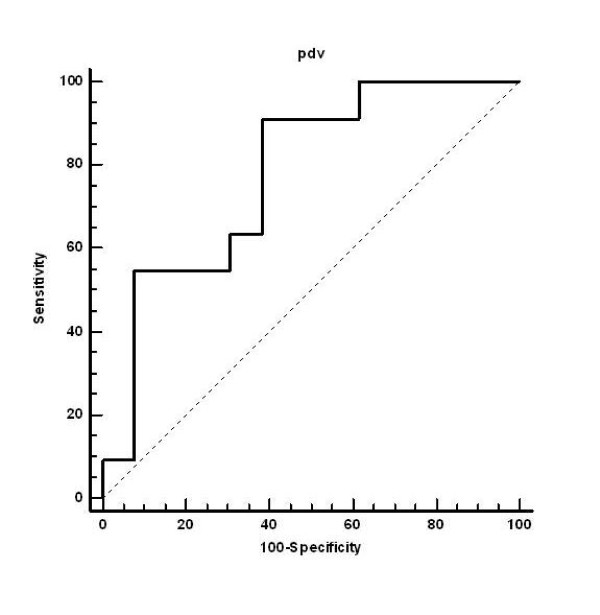
**ROC analysis of peak diastolic velocity (TTDE) and minimum lumen area (IVUS)**.

There was no significant correlation between TTDE and QCA (r = 0.19, p = ns, Figure 1). TTDE measured PDV correlated significantly with IVUS-derived MLA (r = -0.46, p < 0.05, Figure 2) and plaque burden (r = 0.51, p < 0.05, Figure 3). According to the ROC analysis the best cut off for PDV was 112 cm/s (sensitivity, 92%; specificity, 62%) (Figure 4). Correlation data are summarized in Table 3 and 4.

**Table 3:** Correlations of TTDE PDV with QCA and IVUS parameters

Angiography (QCA)		IVUS	
Diameter stenosis	R = 0.19	Plaque burden	R = 0.507
	p = n.s.		p < 0.05

Area stenosis	R = 0.16	Minimum lumen area	R = -0.46
	p = n.s.		p < 0.05

Minimum lumen diameter	R = 0.01	Minimum lumen diameter	R = -0.18
	p = n.s.		p = n.s.

**Table 4:** Correlations between IVUS and QCA parameters

	IVUS Plaque burden	IVUS Minimum lumen area	IVUS Minimum lumen diameter
Angiography	R = 0.61	R = -0.48	R = -0.51

Diameter stenosis	p < 0.05	p < 0.05	p < 0.05

Angiography	R = 0.57	R = -0.41	R = -0.45

Area stenosis	p < 0.05	p < 0.05	p < 0.05

Angiography	R = -0.35	R = 0.64	R = 0.40

Minimum lumen diameter	p = n.s.	p < 0.001	p < 0.05

## Discussion

In our present study we demonstrated, that the measurement of simple resting TTDE might be of additional value in patients with borderline LM lesions.

### Limitations of coronary angiography in LM disease

In many cases, the planar 2D silhouette of the arterial lumen may be unable to accurately define the severity of coronary stenosis, regardless of whether visual or quantitative methods are used. LM has unique anatomical features, which influences the visual and angiographic assessment of lesion severity [29]. LM is a relatively short vessel, and diffuse disease (tubular lesions) often precludes identification of a normal reference segment, leading to underestimation of lesion severity by angiography. Furthermore, the LM is relatively large vessel, therefore non-parallel catheter alignment may lead to an apparent ostial lesion due to contrast streaming, while the overlap of the left anterior descending and left circumflex ostia may obscure the LM bifurcation. Additionally, haziness is often the result of an eccentric plaque seen en face rather than in profile, leading to a reduced volume of contrast dye at the lesion site without producing a stenosis that is quantifiable angiographically. Several studies have shown a poor correlation between IVUS and QCA-derived lumen dimensions in patients with angiographically detected LM stenosis, demonstrating significant intraobserver as well as interobserver variability in the angiographic assessment of the LM [30,31].

### Other invasive methods for the assessment of borderline LM lesions

There is body of evidences regarding the usefulness of both IVUS and fractional flow reserve measurement (FFR) in the evaluation of borderline lesions [11,25,29]. FFR measured by coronary pressure wire provides information regarding functional significance of LM stenosis and is often complimentary to the information provided by IVUS [11]. However, there are still some debates about the determination of criteria for significance. For instance, Jasti et al found that IVUS MLA smaller than 6 mm^2 ^correlated well with FFR < 0.75 [11]. However, Fassa et al. reported a different criterion (MLA by IVUS < 7.5 mm^2^) for a significant LM [10]. In any case, analyses by Sano et al. showed that approximately half of the ambiguous LM narrowing's observed by QCA were hemodynamically significant by IVUS assessment [13]. Our data are in keeping with this observation, since 71% of our patients had significant LM stenosis by IVUS.

### Assessment of the LM with non-invasive methods

There are several non-invasive diagnostic modalities, which can be helpful in assessing LM lesion severity and all these methods can provide important prognostic information, as well. For instance, Dragu et al. found that multidetector computed tomography assessment of LM correlated well with LM IVUS assessed plaque burden [17]. Jasti et. al. found significant correlation between IVUS assessed plaque burden and FFR in cases of ambiguous left main stenosis [11]. Inducible ischemia, detected either during stress perfusion scintigraphy [14,15] or stress echocardiography [16] is also an important factor in the determination of the physiologic severity of LM disease. Coronary flow reserve, assessed by transthoracic Doppler echocardiography has been also extensively studied and proposed as a valuable adjunct in ambiguous cases following coronary angiography [18]. Recently, Anjaneyulu et al. reported that LM stenosis could be assessed by transthoracic echocardiography with an acceptable degree of sensitivity and specificity [21]. Furthermore, Caiati et al [22] have found that the entire LAD can be visualized by the use of contrast-enhanced TTDE and taking a flow acceleration of 0.82% as reference value, for Doppler criterion of significant stenosis, the sensitivity and specificity of the method in identifying all diseased segments was 86 and 95%. However, left main stenosis was not analyzed in their population separately. Therefore, our study expands these findings demonstrating that even in cases with ambiguous coronary angiographic results, a simple measurement of the diastolic flow in the LM by pulsatile Doppler echocardiography can provide additional valuable information and can facilitate the decision making process regarding further treatment.

### Clinical implications

**Figure 5:**
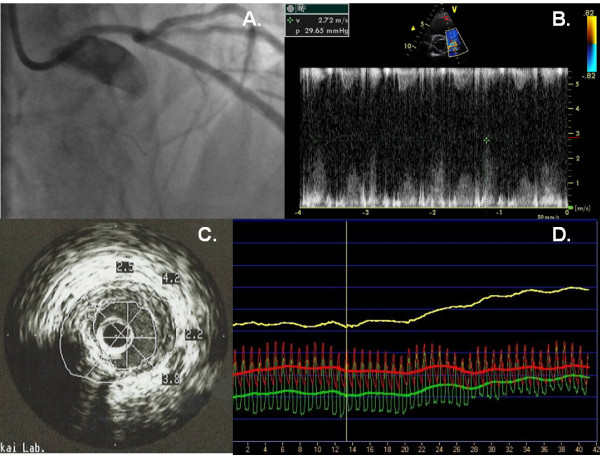
**The imaging sequel of a 64-year old male patient with ambiguous angiography result**. Coronary angiography revealed a tubular 30% stenosis of the LM, and non significant stenosis of the left and right coronary artery (panel A). However, the clinical presentation (severe angina, Canadian classification III-IV) did not corroborate with the angiographical findings, therefore the patient was sent to TTDE, which confirmed very high (272 cm/s) LM resting PDV (panel B). IVUS and FFR were performed, which both confirmed the severity of the LM stenosis (FFR = 0.72; IVUS plaque burden: 65% and LCSA: 4.6 mm2) (panel C and D).

**Figure 6:**
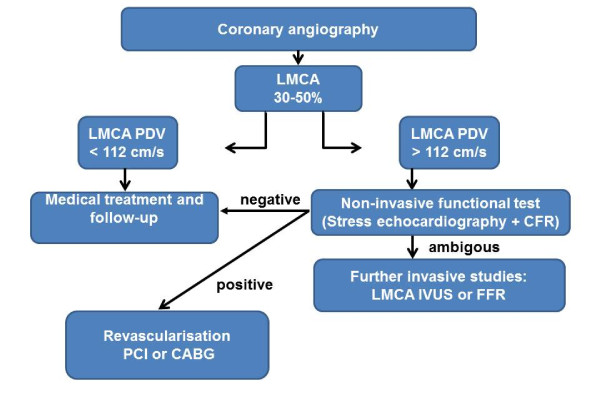
**Left main coronary disease: diagnostic flow chart which incorporates the Doppler echocardiographic parameters**.

Assessing borderline coronary stenosis is an ongoing challenge in invasive cardiology. The optimal techniques are IVUS and FFR measurements, however these techniques are extremely expensive, not widely available, and require special training. Non-invasive techniques like TEE or TTDE using a high frequency transducer are useful for noninvasive evaluation of flow velocity dynamics in the LM [12,20-22,32]. Doppler-TTE is a simple, widely-used, and noninvasive procedure that does not require lengthy preparation and post-examination observation, therefore can be used for routine screening of patients with known ambiguous LM as adjunct to other invasive or non-invasive diagnostic modalities. The cut-off value of 112 cm/sec determined by the ROC analysis demonstrated a good sensitivity of PDV in recognizing hemodynamically significant (determined by IVUS) LM disease, however the specificity of the method was rather low. This can still generate further unnecessary invasive testing's, therefore at this point the PDV cannot considered a standalone method in the evaluation process of the borderline LM narrowing's. A typical borderline LM case is presented in Figure 5 and a possible clinical algorithm is given in Figure 6. Further, large scale studies are needed to establish the exact cut-off value of PDV for routine clinical application.

### Study limitations

The severity of the LM lesion was determined by measuring the flow velocity by Doppler echocardiography, but we don't have evidences how microvascular resistance or other hemodynamic conditions changes the level of PDV. The value of resting Doppler velocity is determined by the severity of the coronary stenosis and other rheological and haemodynamic factors, of which abnormality of the microcirculation may falsely increase the value of PDV, potentially disturbing its assessment. The value of resting PDV can be influenced by haemodynamic variables, as well, such as changes in heart rate, blood pressure and increased contractility. Geometry of the vessel (tortuousity, kinking) and the plaque incidence of the ultrasound beam (eccentric plaque) are also important factors that may influence PDV. The most important limitation of PDV measurement is technical. Very accurate adjustment of the Doppler beam is needed during the measurement, what is in some cases can cause difficulties. For instance, when the orientation of LM is orthogonal in the short-axis view where angle correction exceeded 60 degrees, diastolic flow velocity could be underestimated [21]. Not all echocardiography systems are equipped with the appropriate software and experienced personnel to perform this technique. Today, however, TTE assessment of the LM is a common and accepted and spreading method.

Additional limitation is that our patient group was selected and relatively small, but we focused on the reliability of TTE assessment and not on determining the PDV characteristics of different pathologies. Furthermore, neither functional studies nor coronary flow reserve evaluation were performed in our study population, therefore we don't have data regarding the comparison of the physiologic severity of the lesions and the PDV.

## Conclusion

TTDE evaluation might be a useful adjunct to other invasive and non-invasive methods in the assessment of borderline LM lesions. Further, large scale studies are needed to establish the exact cut-off value of PDV for routine clinical application.

## List of abbreviations

CFR: coronary flow reserve; DS; diameter stenosis; FFR: fractional flow reserve; IVUS: intravascular ultrasound; LM: Left main coronary artery; LAD: left anterior descending artery; MLA: minimum lumen area; PDV: peak diastolic velocity; QCA: quantitative coronary angiography; TTE: transthoracic echocardiography; TEE: transoesophageal echocardiography; TTDE: transthoracic Doppler echocardiography.

## Competing interests

The authors declare that they have no competing interests.

## Authors' contributions

ZR, AP and AV introduced the study idea. AP acquired the ultrasound images. IU and AP performed the off-line analysis. AP helped in the interpretation of the results and statistical analysis. ZR wrote the manuscript, AV added clinical discussion to the manuscript. IU and TF reviewed the manuscript. Finally, all authors read and approved the manuscript.
